# Characterization of Red Blood Cells with Multiwavelength Transmission Spectroscopy

**DOI:** 10.1155/2015/382641

**Published:** 2015-01-12

**Authors:** Yulia M. Serebrennikova, Debra E. Huffman, Luis H. Garcia-Rubio

**Affiliations:** Claro Scientific, LLC., 10100 Dr. Martin Luther King Jr. Street N., St. Petersburg, FL 33716, USA

## Abstract

Multiwavelength transmission (MWT) spectroscopy was applied to the investigation of the morphological parameters and composition of red blood cells (RBCs). The MWT spectra were quantitatively analyzed with a Mie theory based interpretation model modified to incorporate the effects of the nonsphericity and orientation of RBCs. The MWT spectra of the healthy and anemic samples were investigated for the RBC indices in open and blinded studies. When MWT performance was evaluated against a standard reference system, very good agreement between two methods, with *R* ^2^ > 0.85 for all indices studied, was demonstrated. The RBC morphological parameters were used to characterize three types of anemia and to draw an association between RBC morphology and anemia severity. The MWT spectra of RBCs infected with malaria parasite *Plasmodium falciparum* at different life cycle stages were analyzed for RBC morphological parameters. The changes in the RBC volume, surface area, aspect ratio, and hemoglobin composition were used to trace the morphological and compositional alterations in the infected RBCs occurring with parasites' development and to provide insights into parasite-host interactions. The MWT method was shown to be reliable for determination of the RBC morphological parameters and to be valuable for identification of the RBC pathologic changes and disease states.

## 1. Introduction

Numerous diseases result in pathomorphological changes in the red blood cell (RBC) shape, structure, composition, and count [1]. The most common of them is anemia that affects one-third of the world population [2]. Iron deficiency due to malnutrition and infectious diseases like malaria, babesiosis, HIV/AIDS, hookworm infestation, schistosomiasis, and tuberculosis promotes anaemia. Anemia is prevalent in 30% to 90% of patients with cancer [3]. Anemia is a common complication in renal disease patients and can be coupled with echinocytosis and stomatocytosis of RBCs due to hemodialysis oxidative stress [4]. Besides low hemoglobin levels, anemia is frequently associated with abnormalities in the shape of the RBCs such as tear drop, echinocyte, acanthocyte, and sickle cells, along with others [5]. Intracellular parasites of* Plasmodium *spp. induce alterations to the infected RBC morphology and biomechanical properties during their development and eventually rupture the host cells. Pathomorphological changes in the RBCs shape reduce their deformability such that the red cell functions are impaired and the cell survival is shortened [1].

Multiwavelength transmission (MWT) spectroscopy provides substantial information on the physical, chemical, and physiological character of the cells and therefore it is capable of detection and identification of changes in the cells due to diseases [6–8]. Notably, MWT spectroscopy is differentiated by no need of reagents and portability and ease-of-use of instrumentation. With MWT spectroscopy, both absorption and narrow-angle (<2°) forward scattered light are measured simultaneously at broad wavelength range. This optical configuration is different from those commonly used in diffuse transmission spectrometers employing, for example, integrating spheres or similar optical configurations that accept light from a broad range of scattering angles. However, when light from a broad range of scattering angles is being measured simultaneously, the scattering component of the transmitted light is averaged over the scattering angles observed. In spectrometer configurations with narrow acceptance angle that captures light scattered from 0° to 2° observation angles, the averaging effect is minimal and represents scattering component in the measured transmission spectrum.

The absorption component provides information on the chemical composition and the scattering component on the size and structure of the cells. Since scattering properties depend on the refractive index of the measured objects, which, in turn, is governed by chemical composition, simultaneous measurement of both absorption and scattering gives clear advantage over the methods of traditional spectroscopy such as angular light scattering [9, 10] and integrating sphere diffuse transmittance [11, 12] that measure absorption and scattering properties independently. Furthermore, since refractive index of any substance and therefore its absorption and scattering properties vary with wavelength, a single MWT measurement can provide a sufficient number of independent data points to extract information on multiple parameters (in cases of complex composition and/or complex structure) of measured objects. An interpretation model based on an ellipsoidal approximation to the modified Mie theory (MMT) has been found appropriate to extract the information from measured MWT spectra of RBCs [8, 13].

In this paper, we review the method based on MWT spectroscopy coupled with MMT interpretation model for the estimation of the RBC parameters (size, dimensions, and hemoglobin composition) and RBC indices in whole blood (RBC number density, hematocrit, and total hemoglobin). The whole blood samples from 43 healthy donors and from 51 oncology patients undergoing chemotherapy and having cancer related and/or chemotherapy-induced anemia were used for the demonstration. The samples from anemic patients were analyzed blindly. The samples were analyzed with a reference laboratory hematology analyzer (SYSMEX SF-3000) in parallel to the MWT method. In addition, the MWT method was applied for the analysis of laboratory culture samples of RBCs infected with malaria parasite* Plasmodium falciparum*.

## 2. Materials and Methods

### 2.1. Samples

#### 2.1.1. Whole Blood Samples

Whole blood samples from 43 healthy donors were provided by One Blood (St. Petersburg, FL). The samples were collected using lavender top tubes containing K_2_EDTA and analyzed for the complete blood count (CBC) parameters within 24 hours of collection with a laboratory reference system (SYSMEX SF-3000 hematology analyzer). The MWT spectroscopy analysis of the blood samples from healthy donors was conducted in parallel.

Fifty-one EDTA tubes with whole blood from anemic patients were provided by One Blood (St. Petersburg, FL). The samples were collected from the oncology patients at Moffit Cancer Center (Tampa, FL) 1–5 days prior to the analysis. The analyses with MWT and the reference system (SYSMEX SF-3000 hematology analyzer) were conducted in parallel and the results from the reference system were sealed until the end of the study.

#### 2.1.2. *P. falciparum* Culture Preparation

In vitro cultures of the W2 strain of* P. falciparum* were grown in group A+ RBCs at 4% hematocrit in Royal Park Memorial Institute (RPMI) 1640 medium supplemented with 4-(2-hydroxyethyl)-1-piperazineethanesulfonic acid (HEPES), human plasma, and sodium bicarbonate following the described method [7, 8, 14]. Cultures were sampled for the spectrophotometric analysis at ring, early trophozoite, mature trophozoite, and schizont stages of the parasite's asexual life cycle. The parasites' stage was confirmed with microscopic evaluation of Giemsa-stained thin smears.

### 2.2. Spectrophotometric Measurements

All MWT spectra were recorded using a diode array spectrometer (HP 8453 Hewlett-Packard, Palo Alto, CA) having an acceptance angle smaller than 2°. The measurements were conducted with spectra acquisition time of 0.5 sec, signal to noise ratio greater than 10^4^, and 1 nm wavelength resolution. All measurements were conducted at room temperature using a 1 cm pathlength cuvette. Prior to recording a sample spectrum, the spectrometer was zeroed to account for any stray light. To avoid the effect of inhomogeneities in the suspending medium, the background spectrum was taken using a sample from the same batch of phosphate buffered saline (PBS) utilized for the sample measurements.

#### 2.2.1. Whole Blood Measurements

3 *μ*L of a whole blood sample was added to 3 mL of PBS in a spectrophotometric cuvette. The cuvette was gently inverted a few times to ensure homogeneous distribution of the cells. Three replicate spectrophotometric measurements were taken.

#### 2.2.2. Measurements of the RBC Fraction

1 mL of a whole blood sample was centrifuged at 13,000 rpm for 1 min, plasma and buffy coat fractions were aspired, and RBCs were resuspended in 1 mL of PBS. The centrifugation, supernatant aspiration, and resuspension in PBS were repeated 3 times to completely clear the RBC fraction from other blood components. To measure a MWT spectrum, 3–5 *μ*L of RBC fraction was added to 2 mL of PBS in a spectrophotometric cuvette. The aliquot was chosen for the maximal measured optical density to fall within the 0.45–0.6 A.U. range. Prior to the measurement, the cuvette was inverted a few times to ensure homogeneous distribution of the cells. For each sample, five replicate measurements of the RBC fraction of each sample were taken.

#### 2.2.3. Measurements of* P. falciparum* Cultures

1 mL of 4% hematocrit culture containing* P. falciparum* infected RBCs or control noninfected RBCs was centrifuged at 13,000 rpm for 1 min, growth media was aspired, and RBCs were resuspended in 1 mL of PBS. The procedure was repeated 3-4 times until the supernatant was clear. To record a MWT spectrum, 10 *μ*L of suspension was added to 2 mL of PBS in a spectrophotometric cuvette and gently inverted to ensure homogeneous distribution of the cells in the cuvette. For each culture sample, 5 replicate measurements were taken.

### 2.3. Spectral Analysis

#### 2.3.1. Interpretation Analysis of the RBC Spectra

The measured spectra of RBCs from healthy donors, anemic patients, and noninfected control RBCs were interpreted using the homogeneous case of modified Mie theory (MMT) model described in detail in [13]. The model utilized an ellipsoidal approximation to Mie theory to account for the effects of the RBC orientation and nonspherical shape. It has been demonstrated that the ellipsoidal approximation to the RBC shape is quantitatively suitable for the prediction of light attenuation by RBCs in the forward direction [10]. According to the model, the measured optical density was theoretically predicted as a weighted sum of the extinctions of orientation populations:

(1) $$\begin{array}{c} \tau {\left(\lambda \right)}_{\text{calc}}=N_{p}L\sum \omega_{i}\left(\frac{A_{i}}{4}\right)Q_{\text{ext},i}, \end{array}$$

where *τ*(*λ*)_calc_ is the predicted total optical density at given wavelength *λ*, *N*_*p*_ is the total number density of cells, *L* is the pathlength, *ω*_1_ is the weight fraction of the *i*th orientation, and *A*_*i*_ and *Q*_ext,*i*_ are the projected area and extinction, respectively, of the cells at the *i*th orientation. The extinction for each orientation population was a function of the size parameter and refractive index. The size parameter (*χ*) was defined as

(2) $$\begin{array}{c} \chi =\frac{2\pi n_{0}D_{\text{MIE}}}{\lambda }, \end{array}$$

where *n*_0_ is the refractive index of the medium and *D*_MIE_ is the effective Mie diameter of the orientation population computed according to [15] as

(3) $$\begin{array}{c} D_{\text{MIE}}=\frac{a}{g},\\ g={\left(\cos^{2}\psi +\left(\cos^{2}\xi +r^{2}\sin^{2}\xi \right)\sin^{2}\psi \right)}^{1/2}, \end{array}$$

where *r* = *a*/*c* is the ratio of the symmetry axis *a* to minor axis *c* of the ellipsoid and *ψ* and *ξ* are the rotation angles of the ellipsoid's *x*-*z* and *y*-*z* planes with respect to the direction of the incident beam. The complex refractive index of RBCs was computed as the weighted sum of those of hemoglobin and water.

The numerical interpretation procedure consisted of the prediction of spectra as functions of the model parameters, comparison of the predicted and measured spectra, subsequent adjustment of the parameters, new prediction, and comparison. An iterative least squares minimization procedure based on a Nelder-Meade downhill simplex optimization algorithm and variable transformation techniques [16, 17] was used and the model parameters were iterated until convergence (i.e., relative changes in the residual sum of squares (RSSQ) were less than 10^−5^). RSSQ was calculated as

(4) $$\begin{array}{c} \text{RSSQ}=\left(\frac{1}{M}\right)\sum {\left(\tau {\left(\lambda \right)}_{\mathrm{meas},m}-\tau {\left(\lambda \right)}_{\text{calc},m}\right)}^{2}, \end{array}$$

where *M* = 651 and corresponds to the 250–900 nm wavelength range and 1 nm wavelength resolution. The interpretation analysis of a sample spectrum was considered adequate if the RSSQ value was of the order of 10^−3^ or less. The model parameters included RBC length, width, fraction of haemoglobin, and the position angles relative to the light for two orientation populations. The values of the model parameters achieved at the convergence of the predicted and measured spectra became the outcomes of the interpretation analysis. The RBC mean corpuscular volume (MCV) was computed from the estimated length and width. The fraction of hemoglobin was converted to the mean corpuscular hemoglobin concentration (MCHC). The product of MCV and MCHC was mean corpuscular hemoglobin (MCH).

#### 2.3.2. Interpretation Analysis of the Whole Blood Spectra

The measured spectra of whole blood from healthy donors and anemic patients were analyzed for the RBC indices in accordance with (1)–(4). The MWT spectra were predicted using RBC length, width, and fraction of haemoglobin obtained from the interpretation of the corresponding MWT spectra of the RBC fractions as input values. The iterated model parameters were the RBC position angles relative to the light for two orientation populations. In order to avoid the effect of the spectral contribution of blood proteins and cellular components such as platelets and leukocytes, the wavelength range for the interpretation analysis was from 300 to 900 nm. The RBC number density (RBC#) was calculated as the ratio between the measured optical density *τ*(*λ*)_meas⁡_ and the product of the predicted RBC extinction *Q*_ext_(*λ*) and MCV. Hematocrit (HCT) was computed as HCT = MCV ∗ RBC# ∗ 100%. Total hemoglobin (HGB) was computed as HGB = MCH ∗ RBC# ∗ 10^−1^.

#### 2.3.3. Interpretation Analysis of the* P. falciparum* Infected RBC Spectra

The interpretation of the MWT spectra of IRBCs followed (1)–(4) but included a three-layer Mie geometry (i.e., a sphere having three concentric layers) to account for changes in the refractive indices of the IRBC cytosol and the parasite's cytoplasm and organelles. The extinction of each IRBC orientation was computed as weighted sum of the extinctions of three structural groups (Table 1):

(5) $$\begin{array}{c} Q_{\text{ext},i}=\omega_{\text{DV}}Q_{\text{ext},\text{DV}}+\omega_{\text{NU}}Q_{\text{ext},\text{NU}}+\omega_{\text{ORG}}Q_{\text{ext},\text{ORG}}. \end{array}$$

Each structural group was modelled as a three-layer structure such that the outer layer was the IRBC cytosol, the intermediate layer was the parasite's cytoplasm, and the core was a parasite's structural element that provided distinct spectral contribution (digestive vacuole (DV), nucleus (NU), and “average” organelle (ORG)). The validated approximation of additivity of the spectral contributions from the structural groups was used [6]. The real *n* and imaginary *k* parts of the complex refractive index of the *l*th layer were computed as weighted sums of the refractive indices of its compositional constituents:

(6) $$\begin{array}{c} k_{l}=\sum \upsilon_{lj}k_{j}\\ n_{l}=\sum \upsilon_{lj}n_{j}, \end{array}$$

where *υ*_*ij*_ is the weight fraction of the *j*th constituent in the *l*th layer (Table 1). Detailed structure of the IRBC interpretation model is described in [8].

### 2.4. Statistical Analysis

The statistical analysis of the whole blood samples from healthy donors and anemic patients included correlation coefficients, outliers, and bias calculations. Correlation coefficients (*R* ^2^) were obtained using least squares linear regression analysis for each of the RBC CBC indices for the results from the MWT analysis and those from the reference analyzer. Correlation was considered excellent if *R* ^2^ was >0.95, very good if *R* ^2^ was >0.90 and <0.95, good if *R* ^2^ was >0.80 and <0.90, fair if *R* ^2^ was >0.60 and <0.80, and poor if *R* ^2^ was <0.60. The correlation analysis was performed for the whole set of processed samples and on the reduced set without outliers. Outliers were determined on the basis of the difference test. For this purpose, standard deviation (S.D.) of the absolute difference between the results from the MWT analysis and reference analyzer for each RBC CBC index was determined. A data point was classified as an outlier if the absolute difference between the two measurements was greater than the S.D. for two or more parameters. Bland-Altman difference plots were also used to evaluate the agreement between the results from the MWT analysis and reference analyzer for each RBC CBC index. Bias was computed as average over the sample set percentage of the absolute difference between the MWT analysis and reference analyzer values with respect to the reference target value for each RBC index.

## 3. Results and Discussion

### 3.1. Interpretation of the RBC MWT Spectra

**Figure 1:**
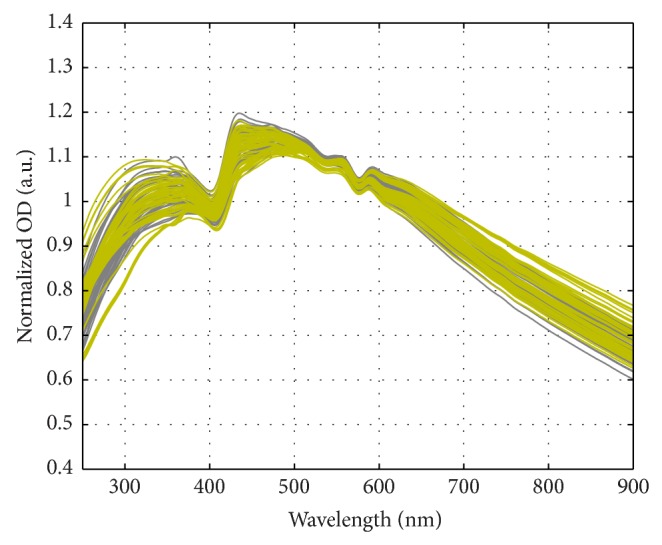
The MWT spectra of RBCs in PBS from healthy donors and anaemic patients.

The variance in the size, dimensions, orientation, and hemoglobin composition of the RBCs resulted in the breadth in the RBC spectral features as can be appreciated in Figure 1. The MMT based interpretation model accounts for all these parameters resulting in excellent prediction of the measured MWT spectra of RBC fractions for samples from healthy donors and anemic patients.

**Figure 2:**
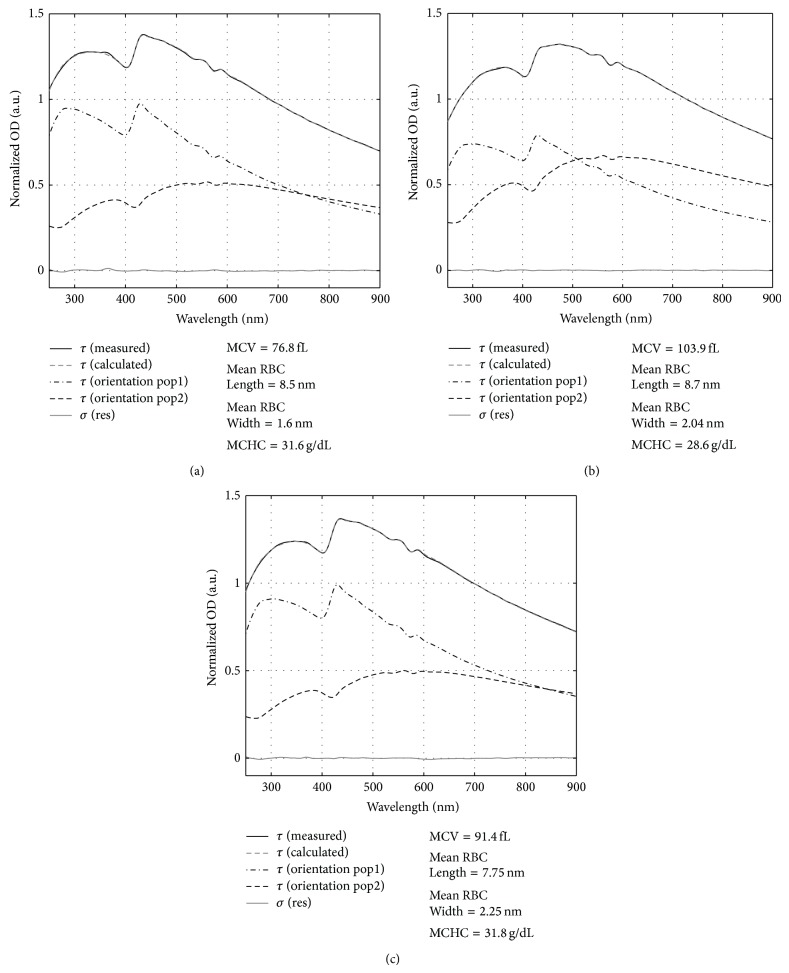
Illustration of the interpretation of the measured MWT spectra *τ* (measured) of RBC fractions. The measured spectra *τ* (measured) are compared to the spectra reconstructed with the MMT based interpretation model *τ* (calculated) and *σ* (res) denotes the difference between the measured and calculated spectra. The reconstructed spectra of two orientation populations are also shown.

The interpretation of the MWT spectra of RBC fractions with MMT model is illustrated with examples in Figure 2. Each figure contrasts a measured MWT spectrum and the corresponding spectrum calculated with the MMT model. The calculated spectrum was predicted as a sum of spectral contributions from two RBC orientation populations also shown in the figures. Excellent agreement between the calculated spectra and the corresponding measured MWT spectra can be noted. The RBC parameters (length and width) derived from the interpretation analysis of MWT spectra and calculated RBC indices are presented in the side panels.

### 3.2. Analysis of RBC Indices

**Figure 3:**
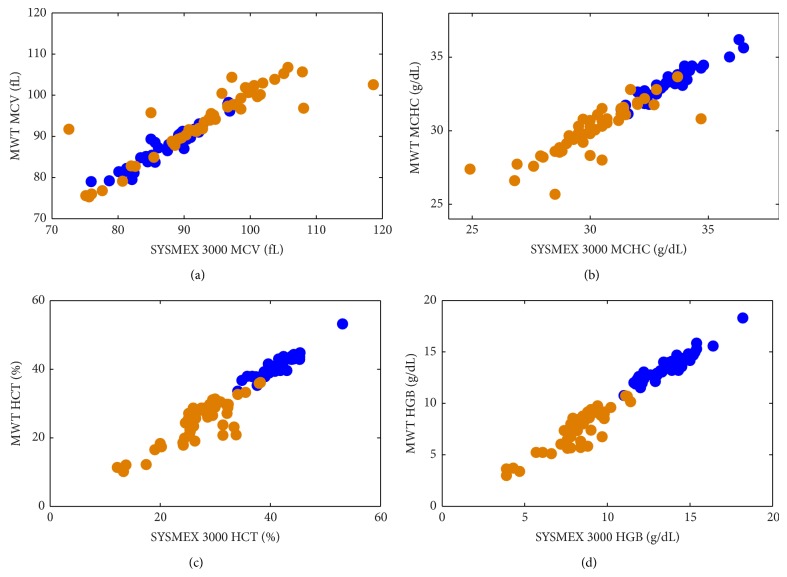
Regressions between selected RBC indices ((a) MCV (fL), (b) MCHC (g/dL), (c) HCT (%), and (d) HGB (g/dL)) obtained through the MMT interpretation of the MWT spectra (*y*-axis) and those obtained with the reference SYSMEX SF-3000 analyzer (*x*-axis) for 43 samples from healthy donors and 51 samples from anemic patients.

The values for selected RBC indices (MCV, MCHC, HCT, and HGB) obtained from the interpretation of the MWT spectra and those from the reference SYSMEX SF-3000 analyzer are compared in Figure 3. The results of the statistical analysis for all RBC indices for the samples from healthy donors are given in Table 2 and for the samples from anemic patients are given in Table 3.

Very good agreement between two methods was achieved for the first set of RBC indices (MCV, MCHC, and MCH) for the samples from healthy donors as the *R* ^2^ values for the regression between the MWT and reference system outcomes were equal to or above 0.90 (Table 2). The statistical analysis of the same RBC index values for the anemic samples showed fair agreement in the whole set of 51 samples (Table 3). However, eight outliers identified with Bland-Altman difference analysis and these data points can be well seen on the scatter plots of Figure 3. Given the age of the samples (it took 1 to 5 days from the sampling and the actual analysis) and the transfer (from the hospital to the laboratory facility), it is not unlikely that these outlier samples were corrupted and/or stored improperly. With eight outliers not considered in the statistical analysis, the correlations between the outcomes of the MWT analysis and SYSMEX SF-3000 analyzer for the remaining 43 samples can be classified as very good (MCHC) to excellent (MCV and MCH) (Table 3). The correlation results for the whole blood RBC indices (RBC#, HCT, and HGB) for healthy donors showed good agreement between two methods (Table 2). The correlations for these indices for the samples from anemic patients showed fair agreement for the whole set of 51 samples but good agreement for the reduced set without data points from previously identified outlier samples (Table 3).

Table 4 gives the summary of the values of RBC indices (MCV, MCHC, MCH, RBC#, HCT, and HGB) for the samples from healthy donors and anemic patients. The mean values for MCHC, RBC#, HCT, and HGB were significantly lower for the samples from anemic patients compared to those from healthy donors (*P* < 0.001, two-tail *t*-test for two samples with unequal variance). In fact, about one-half of the samples from anemic patients had HGB < 8 g/dL that is a threshold for severe anemia [3]. Although the mean MCV value for anemic patients was statistically greater than that for healthy donors (*P* = 0.0005), the range of the MCV values for the anemic patients was greater than that for healthy patients on both lower and higher ends (Table 4; Figure 3(a)).

### 3.3. Analysis of Morphological Parameters of RBCs

**Figure 4:**
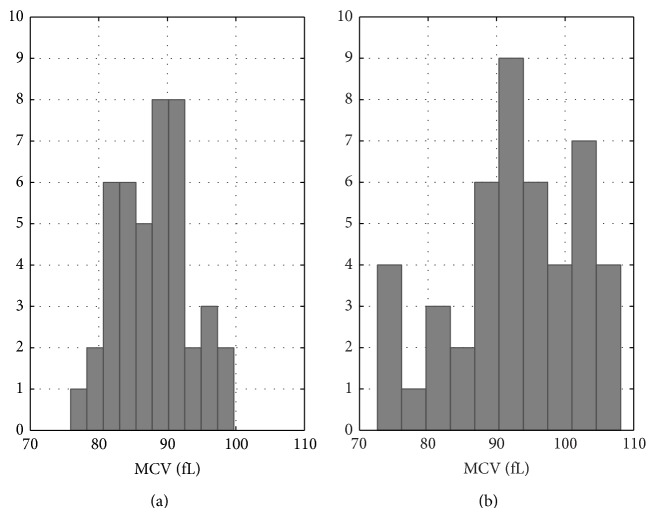
Histogram distribution plots of MCV (fL) index for samples from healthy donors (a) and anemic patients (b).

The MCV distribution for samples from healthy donors approached normal as can be seen in Figure 4(a). On the other hand, the distribution of the MCV values for the samples from anemic patients was more complex with side peaks at low and high MCV ranges (Figure 4(b)). Normocytic anemia, that is, with MCV values within 80–100 fL range, in the case of cancer patients is likely to be indicative of the bone marrow failure [3] and, depending on a variety of factors, including the type and intensity of chemotherapy, occurs in over 60% of cancer patients [18–20]. The bone marrow failure resulted in decreased production of functional RBCs and coupled with cancer related inflammatory response [18] and/or chemotherapy induced [19, 20] increase in destruction of RBCs; blood loss leads to decrease in HGB, RBC count, and HCT. Although hemolysis can also be associated with normocytic anemia, it was not detected in any sample of this study. The peak at smaller MCV values in Figure 4(b) was indicative of microcytic anemia, that is, with MCV values below 80 fL, which is most commonly caused by iron deficiency [3]. Microcytic anemia is not uncommon in otherwise healthy people but with chronic anemia or those with genetic blood disorders such as thalassemia and sideroblastic anemia [3]. The peak at larger MCV values in Figure 4(b) was indicative of macrocytic anemia, that is, with MCV values above 95–100 fL, which is most commonly caused by vitamin B_12_ or folate deficiency [3]. The anemic samples were grouped into three categories on the basis of their RBC MCV values (microcytic with MCV values equal to and less than 82 fL, normocytic with MCV values above 82 fL and below 97 fL, and macrocytic with MCV values equal to and above 97 fL) and the summary of the RBC morphological parameters for these groups along with those for the samples from healthy donors is given in Table 5. It shows apparent morphological differences between the groups. The RBC morphological parameters of samples with normocytic anemia did not differ from those of the samples from healthy patients (*P* = 0.4 for length, *P* = 0.8 for width, *P* = 0.4 for surface area, *P* = 0.8 for sphericity, and *P* = 0.9 for aspect ratio, two-tail *t*-test for two samples with unequal variance) even though they were statistically different in MCHC (*P* < 0.001) and MCH (*P* = 0.005) values as noted above. Microcytic and macrocytic anemic samples had the same range of MCHC values as normocytic anemia samples (*P* = 0.8) but were different in morphology. Both microcytic and macrocytic RBCs were longer with mean length of 8.4 nm than normocytic RBCs with mean length of 8.0 nm (*P* < 0.001). Microcytic RBCs were also thinner with mean width of 1.7 nm than both normocytic and macrocytic RBCs with mean width of 2.2 nm and 2.1 nm, respectively (*P* = 0.004). Consequently, microcytic RBCs had lower sphericity and aspect ratio parameters than normocytic RBCs (*P* = 0.002). Macrocytic RBCs, in turn, had greater surface area than normocytic RBCs (*P* < 0.001). Severe anemia, that is, HGB < 8 g/dL, was not strongly associated with MCV and all three types of RBC morphology, microcytic, normocytic, and macrocytic, were observed in severe anemia samples. Yet, out of 21 samples with severe anemia, 15 were either microcytic or macrocytic (71%) whereas the majority of the samples with nonsevere anemia were normocytic (60%). The RBCs from samples with severe anemia had, on average, lower aspect ratio and sphericity parameters (*P* = 0.002). In this study, the RBC morphology could be associated with the causes of anemia and to some extent with its severity.

### 3.4. Analysis of the Morphological Parameters of* P. falciparum* Infected RBCs

**Figure 5:**
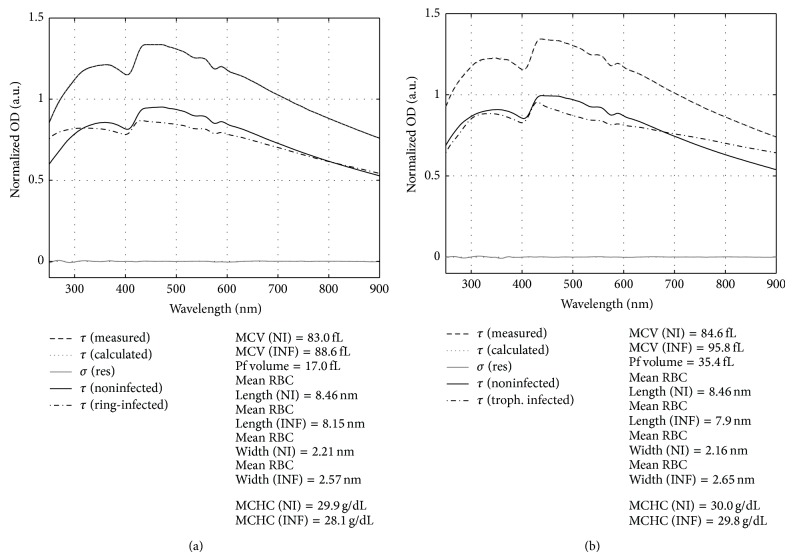
Illustration of the interpretation of the measured MWT spectra *τ* (measured) of the RBC laboratory cultures with 8% (a) and 10% (b)* P. falciparum* parasitemia at trophozoite stage. The measured spectra *τ* (measured) are compared to the spectra reconstructed with the MMT interpretation model *τ* (calculated) and *σ* (res) denotes the difference between the measured and calculated spectra. The reconstructed spectra of the noninfected RBCs were scaled by factors 0.7 (a) and 0.8 (b) whereas the reconstructed spectra of infected RBCs were scaled by factors 8 (a) and 7 (b) for better comparison of the features.

The MWT spectroscopy is highly sensitive to the changes in the size, shape, and composition of RBC that occurred with infection of* P. falciparum* (Figure 5). Each panel in the figure illustrates a MWT spectrum of a laboratory RBC culture containing both noninfected cells and RBCs infected with trophozoite stage* P. falciparum*. Each measured spectrum was interpreted with the MMT model and reconstructed as a weighted sum of the spectral contributions from the noninfected and infected RBCs. These contributing spectra are also contrasted in the panels of Figure 5.

Note that the reconstructed spectra of the noninfected and infected RBCs in Figure 5 were scaled for better comparison of the spectral features (see figure caption for details) and do not show the actual contributions to the total calculated spectra. Significant differences in the spectral features of the malaria infected and noninfected RBCs can be expected as parasites restructure and change the chemical composition of the host cells and these effects can be observed in both panels of Figure 5.

Table 6 summarizes RBC morphological parameters obtained through the interpretation of the MWT spectra of the infected and noninfected control RBC cultures. It shows that all parameters for the noninfected cells used as controls fell within the ranges observed for the RBCs from healthy donors (Table 5). The RBC morphological parameters did not change from the noninfected to ring-stage infected RBCs; however, MCH values reduced due to uptake of hemoglobin by parasites [8]. With further parasites' development, from the ring-stage to early trophozoite stage (<24 hrs), the width of the infected RBCs increased leading to statistically significant increments in the sphericity and aspect ratio parameters (*P* < 0.001, two-tail *t*-test for two samples with unequal variance). Mean RBC volume and surface area of the infected RBCs also increased by ~10% and ~4%, respectively (*P* < 0.001, *t*-test), from the ring-stage to early trophozoite stage. The infected RBC volume, surface area, sphericity, and aspect ratio reached the maxima at mature trophozoite stage (Table 6). Total increments in the RBC parameters at the mature trophozoite stage of* P. falciparum* were 33%, 8%, 22%, and 60% for RBC volume, surface area, sphericity, and aspect ratio, respectively, of those of the noninfected control RBCs. However, the infected RBC volume and surface area decreased significantly, by 31% and ~16%, correspondingly (*P* < 0.001, *t*-test), from trophozoite to schizont stage (Table 6). The infected RBC hemoglobin amount decreased progressively through the cycle of parasite's development by ~90% by schizont stage; the average MCH values were 25.7 pg for noninfected control RBCs and 2.6 pg for infected RBCs at schizont stage.

A long-standing question in the study of malaria is how parasites prevent premature lysis of infected RBCs during the 48 h parasite asexual cycle and the nature of the final release of merozoites from the infected RBCs [21–24]. Although it had long been accepted that the release of merozoites from an infected RBC occurs explosively as the cell becomes more spherical and approaches its critical hemolytic volume toward the end of the parasites' cycle [22, 25], recent measurements of the infected RBC volumes showed little or no increase in the late stage of the parasites' cycle [26, 27]. Our results also strongly supported the increase in the infected RBC volume at trophozoite stage but reduction in the infected cell volume by schizont stage (Table 6). In fact, our results were in good agreement with those of Esposito et al. [27] regarding the changes in the infected RBC volume and surface area from trophozoite to schizont stage. However, Esposito et al. [27] did not observe significant changes in the surface area from ring to trophozoite stage whereas our results showed 8% increment in this parameter for infected RBCs at mature trophozoite stage when compared to noninfected control RBCs (*P* = 0.0003, *t*-test). Although increment in the infected RBC volume was observed, the maximal volume value of 110  ±  12 fL remained well below the critical hemolytic volume value of ~150 fL [25]. The estimated 16% reduction in the surface area of the infected RBCs between the trophozoite and schizont stages was in agreement with estimates made by other methods [27, 28]. Although such reduction in the infected RBC surface area might be a contributing factor to the osmotic fragility of the infected RBC membrane, it is not likely to play a leading role and the structural changes in the infected RBC membrane are caused by parasites [27]. Recent studies suggested that rupture of the host infected RBC at the end of the schizont stage occurs through parasite's controlled spontaneous curvature of and opening of an osmotic pore in the host cell membrane followed by the curling and buckling of the infected RBC membrane [23, 24].

### 3.5. Relationship between Morphological Parameters and RBC Disease State

**Figure 6:**
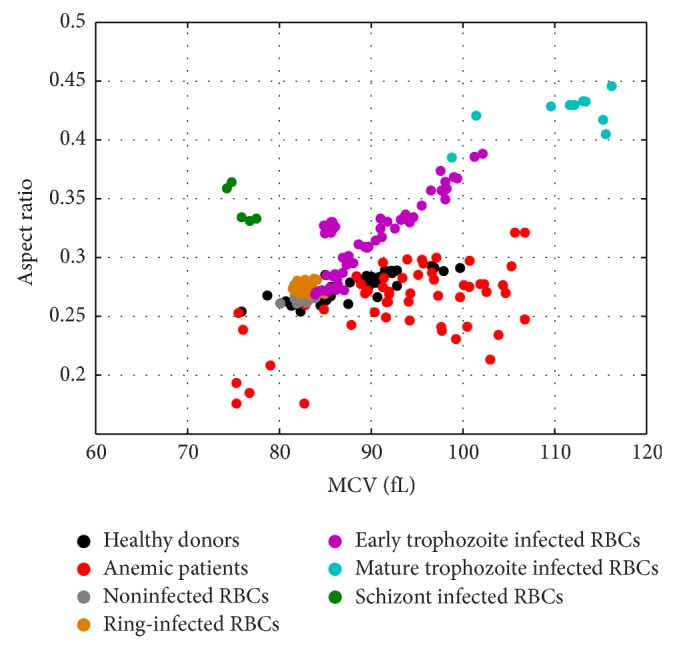
Scatter diagram of RBC aspect ratio versus MCV (fL) estimated from the MMT interpretation of the MWT spectra of RBCs samples from healthy donors, anemic patients, and laboratory RBC cultures infected with* P. falciparum* at different life cycle stages and the corresponding control noninfected RBCs.

Graphic illustration of how diseased RBCs can be readily distinguished from normal healthy RBCs using morphological parameters is presented in Figure 6. It shows scatter diagram of RBC aspect ratio versus MCV for all samples reviewed in this study. There is a clear tight pattern for the distribution of healthy cells on the plot whereas other cells produced considerably greater scatter. Since about a half of the samples from anemic patients had thin RBCs (microcytic or macrocytic), the data points corresponding to anemic patients fell largely below the tight distribution of the data from healthy patients. The data points corresponding to the parasite infected RBCs showed gradual increase in both parameters (volume and aspect ratio) with parasites' growth until mature trophozoite stage. The data points corresponding to the parasite infected RBCs at schizont stage are remarkably different, with low MCV but still high aspect ratio values.

## 4. Conclusions

The aim of this study was to demonstrate that MWT spectroscopy combined with appropriate spectral interpretation techniques provides reliable quantitative estimates of the morphological parameters and composition of normal and diseased RBCs. We showed adequate performance of the MWT method as its estimates of the RBC indices were in very good agreement with those obtained with a reference laboratory system in both open and blinded studies. Morphological parameters extracted from the MWT spectra were extensively applied in RBC recognition to distinguish between normal cells, diseased cells, and parasite infected cells. The MWT analysis of the samples from anemic patients undergoing chemotherapy allowed comprehensive characterization of the morphological features of three types of RBCs in anemia (microcytic, normocytic, and macrocytic). Microcytic and macrocytic RBCs had greater length and surface area than normocytic RBCs. Further, microcytic RBCs appeared as flat discs with significantly lower sphericity and aspect ratio than other RBC types. Analysis of RBC morphology can give insights into the causes of anemia and even its severity. Our results showed the predominance of abnormal RBC shapes (microcytic or macrocytic) in samples with severe anemia (71%). Further, morphologic characteristics of the infected RBCs derived from the interpretation of the MWT spectra allowed tracing the parasite's development and provide insights into parasite-host interactions. Changes in the infected RBC volume, surface area, aspect ratio, and hemoglobin composition during the parasites' development allow for quantitative assessment of the parasites' mediation of the host cells. The MWT method demonstrated in this study determines the RBC morphological parameters and proves to be valuable for identification of RBC pathologic changes and disease states. Given the portability of the MWT instrumentation and the reagentless nature of the method, it could be used in places where standard complex technologies for RBC analysis are not possible.

## Figures and Tables

**Table 1:** The composition of the structural groups of the IRBC interpretation model.

Structural group (shell/core)	Composition of the shell	Composition of the shell	Composition of the core
IRBC body/parasite body/digestive vacuole	Hemoglobin Water	Proteins RNA Water	Hemozoin Hemoglobin Water
IRBC body/parasite body/nucleus			Proteins DNA + RNA + nucleotides Water
IRBC body/parasite body/organelles			Proteins Water

**Table 2:** The slopes and correlation coefficients of the regressions between the MWT analysis and reference analyzer SYSMEX SF-3000 values, bias, and standard deviation (S.D.) for the RBC indices (MCV, MCHC, MCH, RBC#, HCT, and HGB) for the samples from healthy donors.

Stat/parameter	MCV	MCHC	MCH	RBC#	HCT	HGB
Slope (*n* = 43)	1.00	1.00	1.00	0.99	0.99	0.99
*R* ^2^ (*n* = 43)	0.93	0.90	0.91	0.89	0.86	0.89
Bias (*n* = 43)	1.2%	0.9%	1.6%	2.1%	2.3%	2.5%
S.D. (*n* = 43)	0.95	0.40	0.23	0.09	0.79	0.27

**Table 3:** The slopes and correlation coefficients of the regressions between the MWT analysis and SYSMEX SF-3000 values, bias, and standard deviation (S.D.) for the RBC indices (MCV, MCHC, MCH, RBC#, HCT, and HGB) calculated for the full data set of samples from anemic patients (*n* = 51) and for the reduced set that excluded outliers (*n* = 43).

Stat/parameter	MCV	MCHC	MCH	RBC#	HCT	HGB
Slope (*n* = 51)	1.00	1.00	1.00	1.08	1.07	1.07
*R* ^2^ (n = 51)	0.72	0.70	0.70	0.68	0.61	0.65
Bias (n = 51)	2.1%	1.8%	2.5%	10%	11%	11%
S.D. (*n* = 51)	5.0	0.8	1.4	0.3	2.8	0.8

Slope (*n* = 43)	1.00	1.00	1.00	1.05	1.04	1.05
*R* ^2^ (*n* = 43)	0.99	0.93	0.98	0.86	0.86	0.85
Bias (*n* = 43)	0.8%	1.0%	1.3%	4.9%	5.0%	4.4%
S.D. (*n* = 43)	0.6	0.3	0.25	0.2	1.4	0.5

**Table 4:** The mean (±2 S.D.) and the range of values of the RBC indices (MCV, MCHC, and MCH) and whole blood RBC indices (RBC#, HCT, and HGB) for the samples from healthy donors (*n* = 43) and anemic patients (*n* = 43).

Samples	Stat/parameter	MCV (fL)	MCHC (g/dL)	MCH (pg)	RBC# (×10^6^)	HCT (%)	HGB (g/dL)
Healthy donors	Mean ± 2 S.D.	88 ± 11	33.4 ± 2.2	29.4 ± 4.3	4.7 ± 0.9	41 ± 7	13.7 ± 2.8
	Range	76–100	31.3–36.5	24.9–33.8	3.6–6.0	34–53	11.0–18.2

Anemic patients	Mean ± 2 S.D.	94 ± 16	30.2 ± 3.6	28.3 ± 4.6	2.9 ± 1.2	27 ± 11	8.2 ± 3.2
	Range	73–108	24.9–34.7	21.7–34.6	0.09	12–38	3.9–11.4

**Table 5:** The values (mean ± 2 S.D.) of the RBC parameters obtained with the MMT interpretation analysis from the MWT spectra of RBC fractions of samples from healthy donors and anemic patients. The samples from anemic patients were divided into three categories: microcytic with MCV < 82 fL, normocytic with MCV > 82 fL and <97 fL, and macrocytic with MCV > 97 fL.

RBC samples/parameter	MCV (fL)	Surface area (*μ*m^2^)	Length (nm)	Width (nm)	Sphericity	Aspect ratio	MCHC (g/dL)	MCH (pg)
Healthy donors (*n* = 43)	88 ± 11	119 ± 12	8.0 ± 0.4	2.2 ± 0.2	0.71 ± 0.03	0.28 ± 0.02	33.4 ± 2.2	29.4 ± 4.3
Microcytic anemic patients (*n* = 7)	77 ± 5	126 ± 9	8.4 ± 0.4	1.7 ± 0.4	0.61 ± 0.08	0.20 ± 0.06	30.4 ± 3.8	23.6 ± 3.1
Normocytic anemic patients (*n* = 21)	92 ± 6	121 ± 11	8.0 ± 0.4	2.2 ± 0.2	0.71 ± 0.04	0.27 ± 0.04	30.5 ± 3.1	28.1 ± 3.0
Macrocytic anemic patients (*n* = 15)	102 ± 5	130 ± 10	8.4 ± 0.4	2.1 ± 0.2	0.69 ± 0.06	0.26 ± 0.04	29.5 ± 3.3	30.0 ± 2.5

**Table 6:** The values (mean ± 2 S.D.) of the RBC parameters obtained with the MMT interpretation analysis from the MWT spectra of noninfected control and *P. falciparum* infected RBC samples.

RBC samples/parameter	MCV (fL)	Surface area (*μ*m^2^)	Length (nm)	Width (nm)	Sphericity	Aspect ratio	MCHC (g/dL)	MCH (pg)
Noninfected (n = 60)	83 ± 2	124 ± 6	8.2 ± 0.2	2.1 ± 0.1	0.71 ± 0.02	0.27 ± 0.02	31.0 ± 1.2	25.7 ± 0.9
Ring-infected (n = 28)	82 ± 3	124 ± 3	8.2 ± 0.1	2.2 ± 0.1	0.70 ± 0.02	0.27 ± 0.02	31.4 ± 2.4	22.4 ± 1.7
Early troph. infected (n = 40)	91 ± 10	128 ± 6	8.2 ± 0.3	2.6 ± 0.5	0.76 ± 0.05	0.32 ± 0.05	29.1 ± 7.0	19.6 ± 5.2
Late troph. infected (n = 10)	110 ± 12	130 ± 8	7.9 ± 0.2	3.3 ± 0.3	0.85 ± 0.03	0.42 ± 0.03	17.6 ± 1.8	9.1 ± 4.0
Schizont infected (n = 5)	76 ± 3	110 ± 6	7.5 ± 0.3	2.6 ± 0.1	0.79 ± 0.03	0.34 ± 0.03	20.4 ± 1.4	2.6 ± 0.6
